# WITHDRAWN: A New Aneurysm Rupture-Prone Marfan Mouse Model with FBN1Q2467X Nonsense Mutation Reveals Adventitial Inflammation

**DOI:** 10.1101/2025.09.23.678101

**Published:** 2025-10-20

**Authors:** Shichao Wu, Jiawei Zhao, Alejandro Ponce, Lucynda Pham, Daniel Xie, Donghong Ju, Francis Hernandez, Sean Jones, Clair Li, Charles S. Chung, Dragana Komnenov, Noreen F. Rossi, Zhe Yang, Maozhou Yang, Hui Li, Youming Xie, Kang Chen, Kezhong Zhang, Li Li

## Abstract

The authors have withdrawn this manuscript because they plan to resubmit a revised version with a refined study scope, excluding the high-fat-diet (HFD) data, and to prepare a separate manuscript focusing on HFD-induced mechanisms of aneurysm progression in the Marfan mouse model. Therefore, the authors do not wish this work to be cited as reference for the project. If you have any questions, please contact the corresponding author.

