# Supplementary material for: A Marfan-Associated FBN1 Nonsense Mutation Mouse Model Reveals Adventitial Inflammation During Aneurysm Progression
Source: bioRxiv. 2025 Nov 25:2025.09.23.678101. Originally published 2025 Sep 25. Preprint. [Version 4] doi: 10.1101/2025.09.23.678101 (PMC12485906; doi:10.1101/2025.09.23.678101)
Supplement: Supplement 1 [file NIHPP2025.09.23.678101v4-supplement-1.pdf]

bioRxiv preprint doi: https://doi.org/10.1101/2025.09.23.678101; this version posted November 25, 2025. The copyright holder for this preprint (which was not certified by peer review) is the author/funder, who has granted bioRxiv a license to display the preprint in perpetuity. It is made available under aCC-BY 4.0 International license.

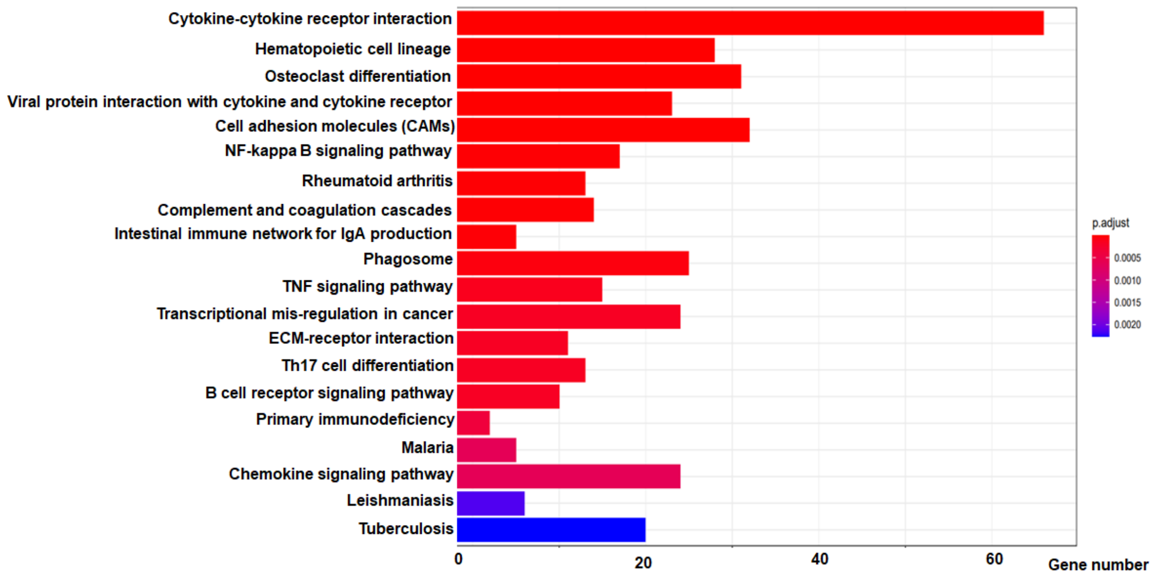

Figure S1. The KEGG analysis of RNA-seq data. The KEGG pathway enrichment analysis was generated with the significantly differentially expressed genes (*P*<0.01) in the RNA-seq of FBN1Q2469X/Q2469X mice and WT. Top 20 KEGG pathways are shown with bar plot: the color represent the adjusted p value of enrichment the KEGG pathways; X axis represents the gene number involved in the KEGG pathways.

bioRxiv preprint doi: https://doi.org/10.1101/2025.09.23.678101; this version posted November 25, 2025. The copyright holder for this preprint (which was not certified by peer review) is the author/funder, who has granted bioRxiv a license to display the preprint in perpetuity. It is made available under aCC-BY 4.0 International license.

Table S1. *FBN1* is highly conserved between human and mouse

FBN1	**Human**	**Mouse**
**Chromosome**	**15**	**2**
**Gene size**	**237.4kb, 66 exons**	**66exons**
**Exons/ATG/TAA**	**66/2/66**	**66/2/66**
**c.7399C>T transition**	**In Exon 59 (CAG-TAG)**	**In Exon 60 (CAG-TAG)**
**mRNA (NCBI)**	**11756bp**	**9847bp**
**Protein**	**2873aa (350KD)**	**2873aa**
**Protein identity**	**2765/2873= 96.24%**	
**Protein similarity**	**2834/2873= 98.64%**	
**a nonsense mutation**	**Q2467X**	**Q2469X**
