# Supplementary material for: A New Aneurysm Rupture-Prone Marfan Mouse Model with FBN1Q2467X Nonsense Mutation Reveals Adventitial Inflammation
Source: bioRxiv. 2025 Sep 25:2025.09.23.678101. Preprint. [Version 1] doi: 10.1101/2025.09.23.678101 (PMC12485906; doi:10.1101/2025.09.23.678101)
Supplement: Supplementary file 1 [file NIHPP2025.09.23.678101v1-supplement-1.pdf]

bioRxiv preprint doi: https://doi.org/10.1101/2025.09.23.678101; this version posted September 25, 2025. The copyright holder for this preprint (which was not certified by peer review) is the author/funder, who has granted bioRxiv a license to display the preprint in perpetuity. It is made available under aCC-BY 4.0 International license.

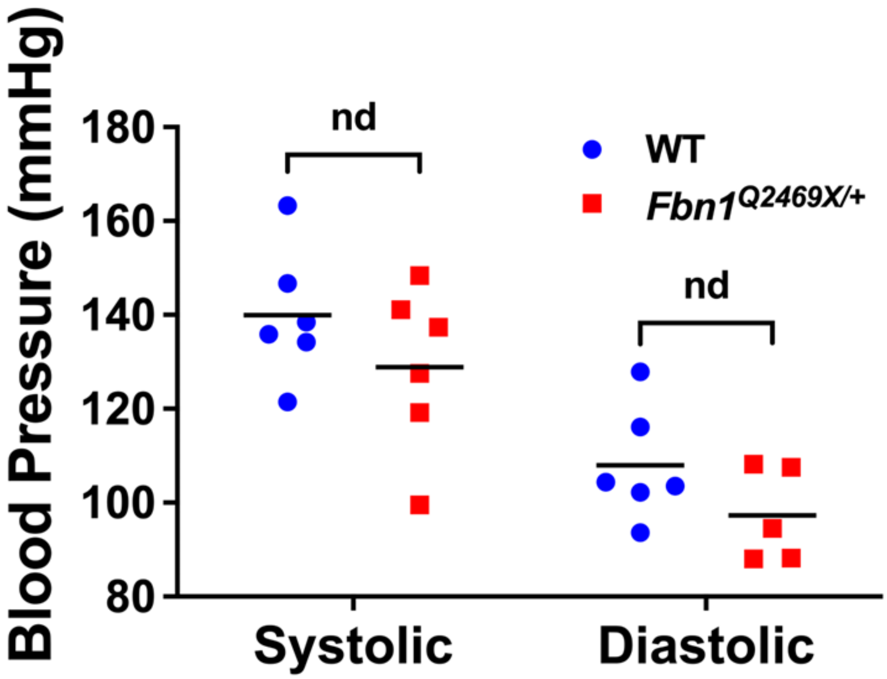

Figure S1. The blood pressure analyses in WT and *FBN1*$^{Q2469X/+}$ mice. There are no statistic difference between WT and *FBN1*$^{Q2469X/+}$ mice although the *FBN1*$^{Q2469X/+}$ mice show slightly decrease in blood pressure. n=10 both male and female mice are used.

bioRxiv preprint doi: https://doi.org/10.1101/2025.09.23.678101; this version posted September 25, 2025. The copyright holder for this preprint (which was not certified by peer review) is the author/funder, who has granted bioRxiv a license to display the preprint in perpetuity. It is made available under aCC-BY 4.0 International license.

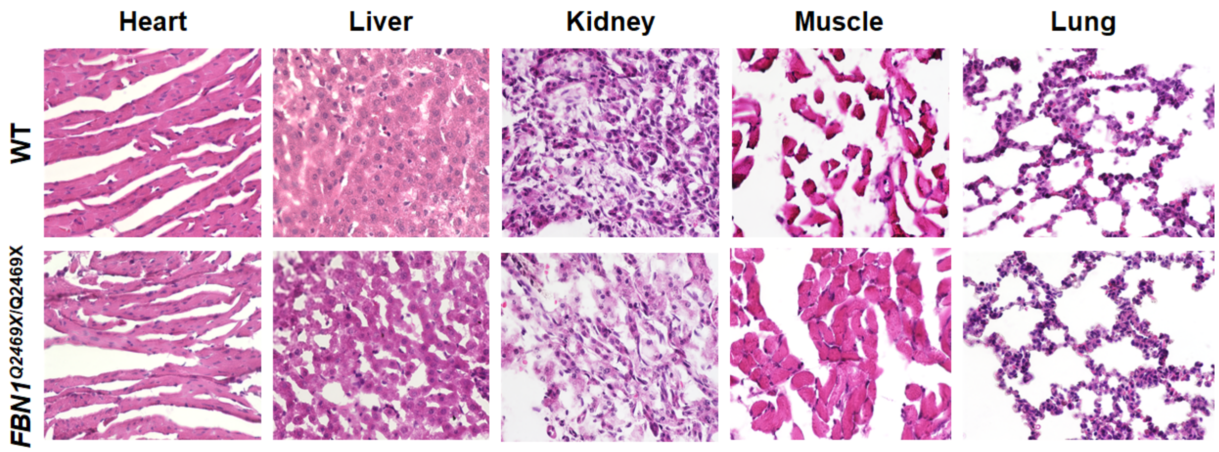

Figure S2. Histopathological analysis of systemic organs in WT and *Fbn1*$^{Q2469X/Q2469X}$ mice. Representative hematoxylin and eosin (H&E) staining of heart, liver, kidney, skeletal muscle, and lung tissues from wild-type (WT) and homozygous *Fbn1*$^{Q2469X/Q2469X}$ mice. Images were acquired at identical magnification to allow direct comparison. No major pathological abnormalities were observed in these organs, likely reflecting the young age of the pups at day 13.

bioRxiv preprint doi: https://doi.org/10.1101/2025.09.23.678101; this version posted September 25, 2025. The copyright holder for this preprint (which was not certified by peer review) is the author/funder, who has granted bioRxiv a license to display the preprint in perpetuity. It is made available under aCC-BY 4.0 International license.

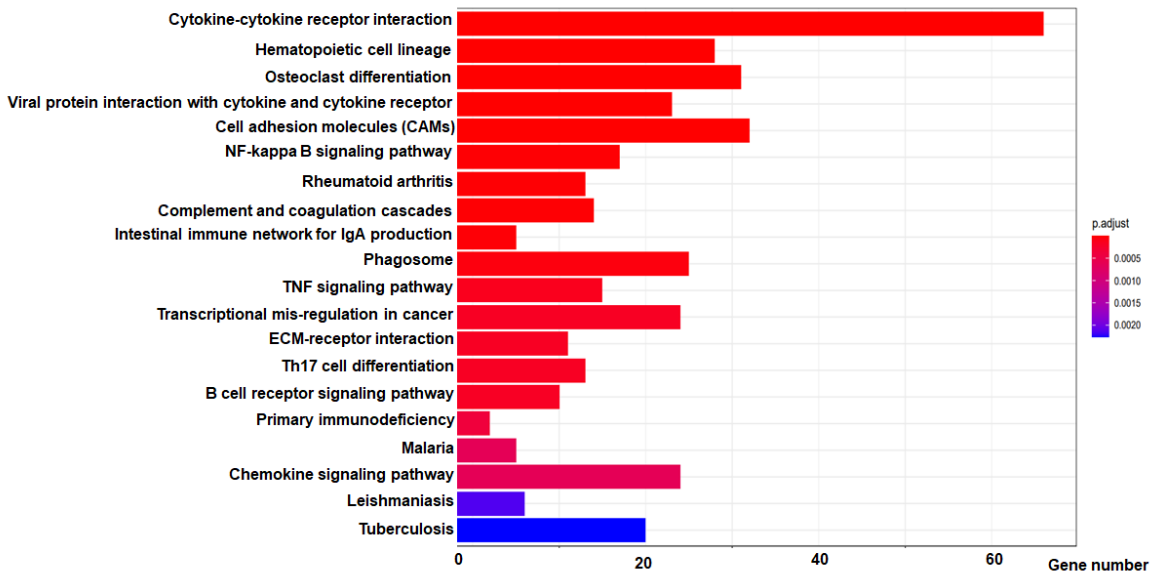

Figure S3. The KEGG analysis of RNA-seq data. The KEGG pathway enrichment analysis was generated with the significantly differentially expressed genes (*P*<0.01) in the RNA-seq of FBN1Q2469X/Q2469X mice and WT. Top 20 KEGG pathways are shown with bar plot: the color represent the adjusted p value of enrichment the KEGG pathways; X axis represents the gene number involved in the KEGG pathways.

bioRxiv preprint doi: https://doi.org/10.1101/2025.09.23.678101; this version posted September 25, 2025. The copyright holder for this preprint (which was not certified by peer review) is the author/funder, who has granted bioRxiv a license to display the preprint in perpetuity. It is made available under aCC-BY 4.0 International license.

***Table S1 FBN1* is highly conserved between human and mouse**

FBN1	Human	Mouse
Chromosome	15	2
Gene size	237.4kb, 66 exons	66exons
Exons/ATG/TAA	66/2/66	66/2/66
c.7399C>T transition	In Exon 59 (CAG-TAG)	In Exon 60 (CAG-TAG)
mRNA (NCBI)	11756bp	9847bp
Protein	2873aa (350KD)	2873aa
Protein identity	2765/2873= 96.24%	
Protein similarity	2834/2873= 98.64%	
a nonsense mutation	Q2467X	Q2469X
